# Lock-in vibration retrieval based on high-speed full-field coherent imaging

**DOI:** 10.1038/s41598-021-86371-3

**Published:** 2021-03-29

**Authors:** Erwan Meteyer, Silvio Montresor, Felix Foucart, Julien Le Meur, Kevin Heggarty, Charles Pezerat, Pascal Picart

**Affiliations:** 1 Laboratoire d’Acoustique de l’Université du Mans, LAUM CNRS 6613, Le Mans Université, Avenue Olivier Messiaen, 72085 Le Mans Cedex 09, France; 2 Institut d’Acoustique, Graduate School, CNRS, Le Mans Université, Avenue Olivier Messiaen, 72085 Le Mans Cedex 09, France; 3 ENSIM, Ecole Nationale Supérieure d’Ingénieurs du Mans, rue Aristote, 72085 Le Mans Cedex 09, France; 4 Département d’Optique, IMT-Atlantique, Technopole Brest-Iroise, CS 83818, 29285 Brest, France

**Keywords:** Optical sensors, Imaging and sensing, Optical metrology, Mechanical engineering, Imaging techniques, Acoustics

## Abstract

The use of high-speed cameras permits to visualize, analyze or study physical phenomena at both their time and spatial scales. Mixing high-speed imaging with coherent imaging allows recording and retrieving the optical path difference and this opens the way for investigating a broad variety of scientific challenges in biology, medicine, material science, physics and mechanics. At high frame rate, simultaneously obtaining suitable performance and level of accuracy is not straightforward. In the field of mechanics, this prevents high-speed imaging to be applied to full-field vibrometry. In this paper, we demonstrate a coherent imaging approach that can yield full-field structural vibration measurements with state-of-the-art performances in case of high spatial and temporal density measurements points of holographic measurement. The method is based on high-speed on-line digital holography and recording a short time sequence. Validation of the proposed approach is carried out by comparison with a scanning laser Doppler vibrometer and by realistic simulations. Several error criteria demonstrate measurement capability of yielding amplitude and phase of structural deformations.

## Introduction

In many fields of applications such as ground transportation, naval or aeronautics, structural vibrations are linked to the notions of acoustic comfort, in the sense of noise radiated by vibrating sources, and to mechanical reliability. Those vibrations may be generated by different solicitations which can originate from mechanical, acoustic, aerodynamic, magnetic excitation, etc. The identification of the vibratory sources and/or the understanding of the vibratory propagation phenomena are often realized with the analysis of operational responses, that is the analysis of the vibratory field resulting from the sources in the practical context. The operational responses permit, for example, the analysis of vibratory transfer paths or modal analysis of the structure. The vibration field is the basic input data for such methods. There are several experimental means with more or less sophisticated protocols to provide vibration fields. Vibration analysis entered the industrial field in the 1970s with the aim, on one hand, to monitor the state of health of the systems^1–3^ and on the other hand, to reduce nuisances caused by these vibrations. For this purpose, sensors have been specially designed for these measurements, such as the accelerometer and a few years later, the laser vibrometer. The accelerometer is a widely used sensor in the academic and industrial worlds. Its strengths are its reliability, robustness, small size, low weight, sensitivity and large bandwidth. It yields point-wise measurements of the vibratory field at the point where it is fixed to the structure.

In order to obtain collection of data points related to the vibration field, it is therefore necessary to repeat the measurement by displacing the sensor, or to increase the number of sensors while increasing the overall complexity of the set-up. In addition, any accelerometer having a certain mass, the behavior of the structure can be modified. In the majority of applications, the mass is chosen so that the structure does not suffer from any perturbation. Nevertheless, in the case of a light weight structure, accelerometers are intrusive. The significant progresses in laser technology and instrumentation lead to the development of contact less measurements by laser Doppler vibrometer. The basic principle is the same as the well known Doppler effect and yields velocity measurements^4–7^. The main interest of the laser vibrometer is for measuring the vibratory field without any contact and without any intrusion at the surface of the structure. In addition, the development of scanning laser Doppler vibrometers gives the possibility to obtain a collection of data points at the inspected surface^8–11^. For example, scanning of a line^8,9^ with 256 points along one line up to 80 kHz^8^, use of holographic optical elements associated with one CMOS sensor (vibration measured up to 100 kHz^10^ ), use of frequency multiplexing (20 points with $5 \times 4$ beams^11^), or use of three acousto-optical devices and a single high-speed photodetector ($5 \times 4$ beams with a rate of 500 Msamples/s^12^). Although these approaches yield a set of independent measurements at several points on the surface, the number of simultaneous measurements is reduced. This requires a repetition of the measurement, and therefore, the use of a controlled and repeatable excitation source. Various evolution emerged in recent years, with the 3D extension of the scanning laser Doppler vibrometer, in order to measure the 3 components of the vibration field, and possible coupling with robots^13^. Such station is very power-full for complex structure analysis, but it requires heavy financial investment and loses its easy use in terms of tool mobility.

The simultaneous collection of a large amount of data points at the surface of dynamic structures can be obtained by other existing approaches around the stereo-correlation of images^14–16^. This yields large field measurements for movements or deformations with high amplitude and it has undeniable assets: non-intrusive, full-field, and easy to implement. In the context of vibration measurement, 3D vision methods with high-speed cameras, as part of dynamic photogrammetry, have been adapted to measure structural vibrations^17–19^. As high-speed cameras are expensive and require accurate synchronization, the unconventional single-camera pseudo-stereo system was proposed, for which the camera sensor is split into two halves thus generating two virtual cameras. The interest of Digital Image Correlation coupled to this pseudo-stereo set-up and a single high-speed camera has recently been used to measure the vibrations of a plate by comparing the results to a reference technique^18^. Since the method considers intensity changes in images, it is less sensitive than laser Doppler vibrometers and targets high amplitude displacements. It follows that this measurement technique is adapted for low-frequency measurements. Some recent works^20^ have been carried out in order to obtain more accurate results in the high-frequency range.

Recently, authors proposed to use deflectometry which is an optical method developed for the full-field measurement of surface slopes, so that no scan operation is required^21,22^. Deflections and curvatures that are often needed for calculations in mechanics can be obtained, respectively, by a single spatial integration and differentiation of the measured slopes. As a result of full-field data recording, the acquisition time is independent of the number of measurement points, enabling dense spatial measurements to be performed in a fraction of the time required for a classical scanning vibrometer. With the use of high-speed cameras, it follows that stationary and transient excitation cases can be analyzed^23,24^. Dynamic mechanical point loads were measured this way in^25,26^. For example, in^27^, pressure reconstructions of an impinging air jet on a flat plate were obtained for mean distributions. Nevertheless, the use of deflectometry requires specific surface preparation so that the surface behaves like a polished flat mirror. This limits the applicability of the technique for naturally rough and non polished surface. Recent work^28^ showed that the use of both infrared lightening and ultra-fast infrared cameras may overcome such limitation.

Full-field evaluation of surface deformation, shape and vibration can be obtained with coherent imaging. It requires expanded coherent laser beams to produce interferences by mixing with an other controlled laser beam. The coherent imaging approach yields a high density of data points and includes variety of approaches such as shearography, speckle interferometry and digital holography^29,30^. The application of holography to vibration analysis was proposed by Powell and Stetson^31,32^ with demonstration of the time-average principle to get direct visualization of the vibration map. Since such process induces the loss of the phase of the vibration, more quantitative methods were developed with the stroboscopic illumination^33–37^ and the laser-pulse regime^38,38–43^. As examples, these approaches were applied to vibration of micro-membranes^37^, modal analysis^40,41^, determination of structural intensity^41^, the observation of acoustic waves^40–42^, the high amplitudes of self-oscillations of a clarinet reed^35^, or also shocks^43^. Although providing quantitative data, the recording process required complex operations such as phase-shifting and laser pulse triggering. More recently, the use of high-speed sensors permitted to acquire holographic data at the time-evolution of the studied phenomena^42,44–49^. The advantage is that the optical assembly is considerably simplified as it does not require a pulsed laser, double pulse laser, or any generation of strobe light pulses. Impressive demonstrations were proposed when applying the methods to life sciences^44,45,47^ or sound measurements^48^.

In reference^48^, authors presented a coherent imaging technique based on space-division multiplexing, known as parallel phase-shifting digital holography. In their paper, they pointed out that, as a general rule, vibration displacements are smaller at high frequencies than at low frequencies and that digital holography requires to be accurate in the direction of the depth to be used for high-speed 3D vibrometry. In addition, they wrote that the accuracy of measurement of conventional holographic systems is not sufficient for precise 3D measurement of fast phenomena because of the superposition of noise terms like the 0${\mathrm{th}}$ order diffraction waves and the conjugate images. In order to bypass that, authors^50,51^ proposed a Fourier filtering method to extract the desired term from recorded holograms because the desired term can be easily separated from parasitic noise in the Fourier domain when the spatial resolution of holograms is sufficiently high. However, the spatial resolution of high-speed sensors generally decreases as the frame rate increases and their large pixels $(\sim 18-20 \upmu m)$ induces an additional reduction in the resolution, making any Fourier filtering difficult to implement. In this paper, we report contact-less large field vibrometry with high-speed coherent imaging based on on-line digital holography, yielding full-field amplitude and phase vibration measurements. Whereas on-line digital holography suffers from the overlap of the diffraction order^49^, we discuss and apply a vibration retrieval algorithm that yield accurate measurements.

The goal of the paper is to propose an adapted for full-field operational vibration reconstruction adapted to the high density of measurement points from digital holography which permits the direct measurement of an extended area with single-shot experiments. The paper compares the ability of the proposed method to reconstruct full-field operational vibrations and LDV measurements used as a reference. The outputs from the proposed processing scheme can be used not only for modal analysis but also for multiple post-treatments: for material characterization (for example with the force analysis technique^52^) or source identification (reconstruction methods compatible with full-field data such as force analysis technique^53^ or virtual field method^54^ for example).

The algorithm restores the operational displacement fields in terms of amplitude and phase of a structure submitted to harmonic excitation. It only requires a short time sequence of digital holograms for the reconstruction of the vibration data. Validation of the proposed approach is carried out by comparison with a scanning laser Doppler vibrometer^55^ and by realistic simulations. Several error criteria demonstrate its ability to yield amplitude and phase of structural deformations.

## Results

**Figure 1:**
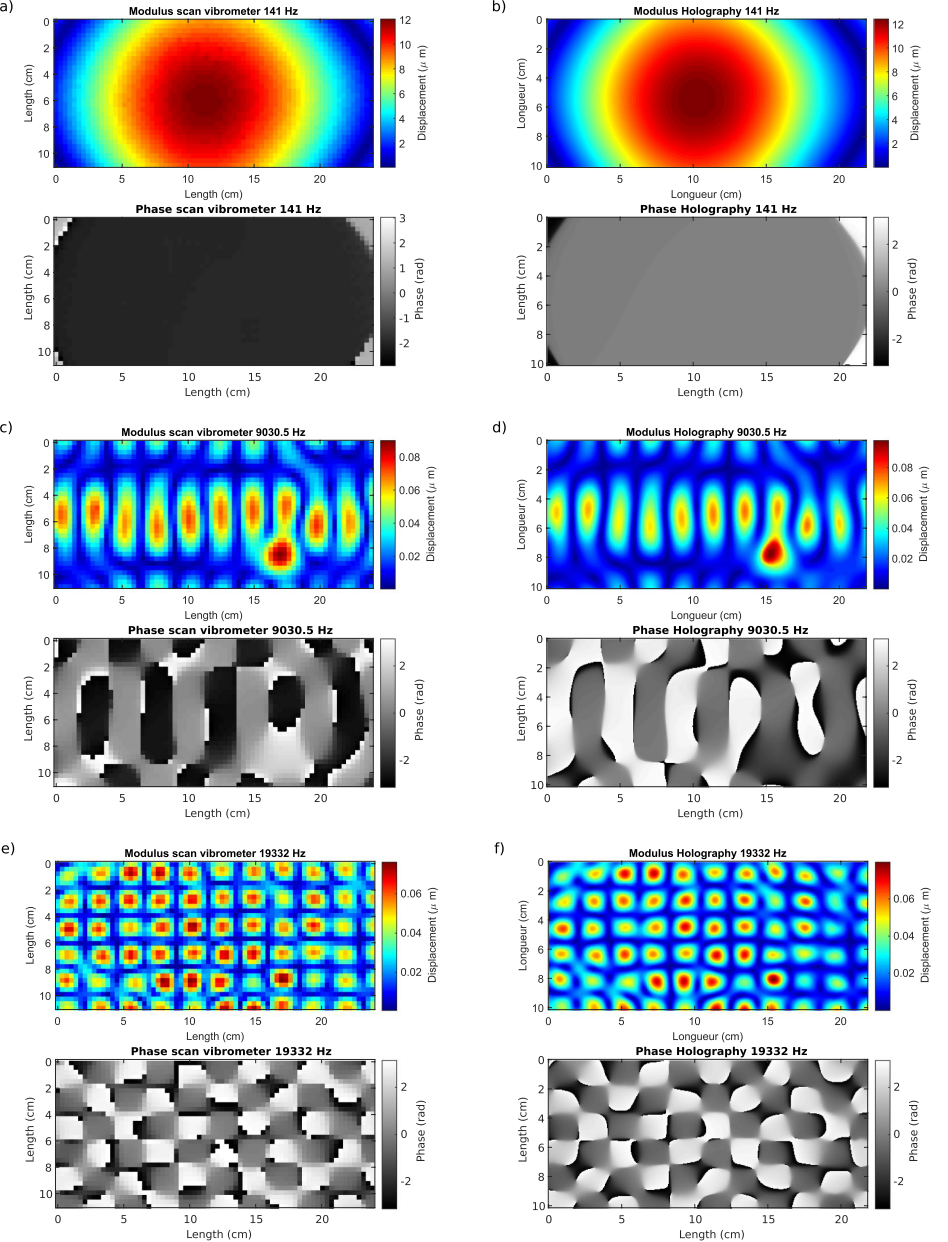
Amplitude and phase of the operational displacement fields of the structure measured with scanning point-wise laser Doppler vibrometer at **(a)** 141 Hz, **(c)** 9030.5 Hz, **(e)** 19332 Hz, and coherent imaging at **(b)** 141Hz, **(d)**, 9030.5Hz, **(f)** 19332Hz.

The significant advantage of the high-speed vibrometry imaging technique discussed here is that it yields a full and large field, high spatial resolution, contact-less, and non-intrusive amplitude and phase measurement of any structural vibration. The retrieval algorithm, called *VibMap* in this paper, permits to retrieve the operational displacement fields of structures with data provided from on-line digital holography. In order to demonstrate this, sequences of holograms were recorded under sinusoidal excitation of a mechanical structure composed of a steel plate with dimensions $30 \times 30$cm$^2$ and 2mm thick. The structure is clamped at one of its lower edge and excited by an electro-mechanical loudspeaker. Several excitation frequencies were considered in the vibroacoustic range [10 Hz–20 kHz]. Figure 1 shows a comparison of amplitude and phase of the structural vibrations measured both with a scanning laser Doppler vibrometer (considered here as the reference method both for comparison of the amplitude and phase data) and the coherent imaging approach for three frequencies at 141 Hz, 9030.5 Hz and 19,332 Hz. The measurements are obtained at frame-rate 40kHz with respectively 10,000, 3400, and 2000 holograms for 141 Hz, 9030.5 Hz and 19332 Hz, corresponding to respectively of 35.25, 767.6 and 966.6 vibration periods. Qualitatively, to the naked eye, the operational displacement fields obtained from both measurement techniques are very close showing an overall good agreement. Measurements from laser scanning vibrometer were carried out with a Polytec PSV-500 Scanning Vibrometer and provide $65 \times 65=4225$ data points whereas those from holography include $463 \times 454=210202$ data points.

**Figure 2:**
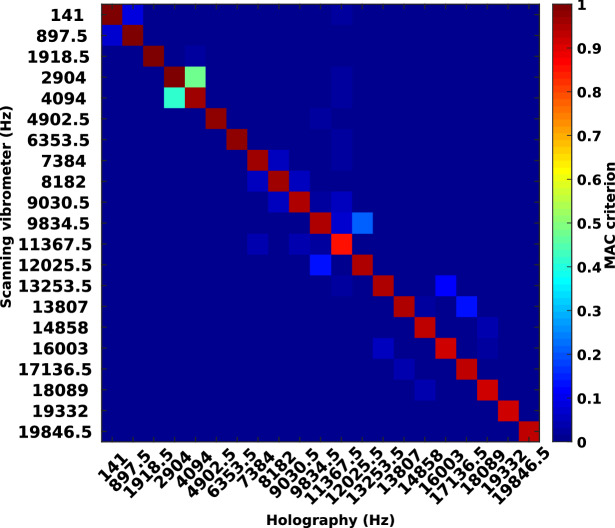
MAC criterion between scanning point-wise vibrometer and coherent imaging measurements for the set of 21 excitation frequencies from 141 to 19,846.5 Hz.

In order to get more quantitative appraisal of the similarity of both results, a comparison was performed by considering the MAC criterion for 21 resonance frequencies covering the range $[0-20]$ kHz. The MAC for the holographic and scanning vibrometer measurements is displayed in Fig. 2. Each frequency was chosen close to resonance frequencies, approximately every 1 kHz.

**Figure 3:**
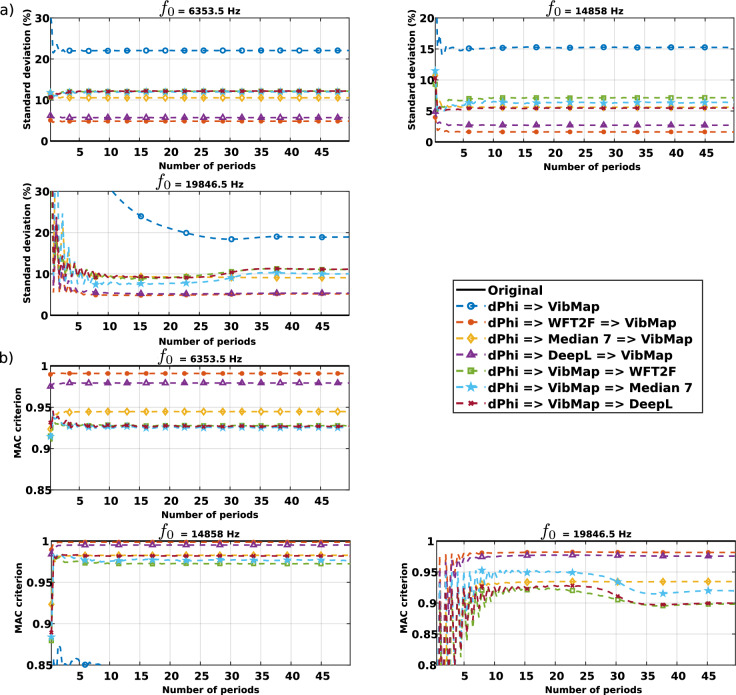
Comparison for each processing scheme applied to simulated data, **(a)** standard deviation for frequencies 6353.5 Hz, 14,858 Hz, 19,846.5 Hz, **(b)** MAC criterion for the same frequencies.

**Figure 4:**
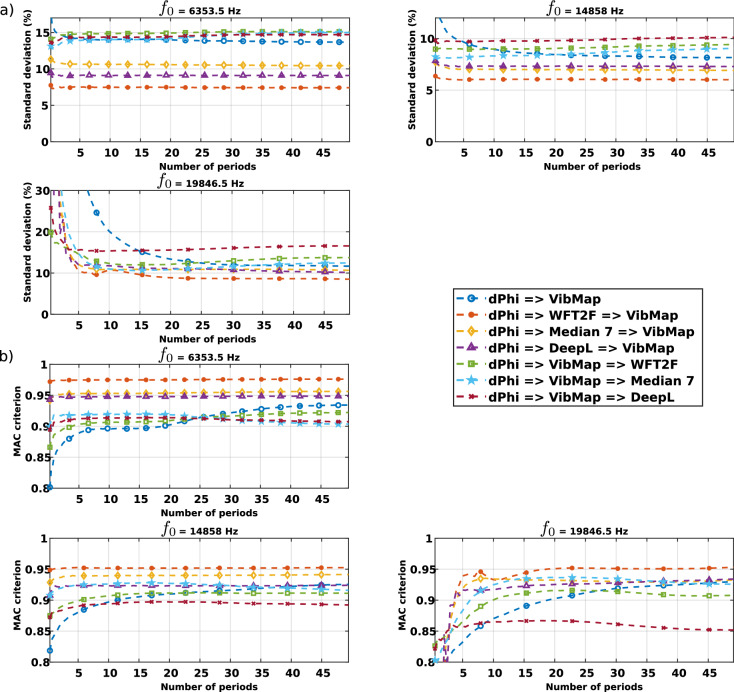
Comparison, for each processing scheme applied to measured data, with, **(a)** the standard deviation for frequencies 6353.5 Hz, 14,858 Hz, 19,846.5Hz, **(b)** MAC criterion for the same frequencies.

Since the measurements obtained from holography are influenced by the full processing chain, this may lead to errors and bias in the final result. In order to quantitatively appraise the impact of the processing of the Doppler phase from the sequence of digital holograms, a realistic simulation was carried out. The displacement field calculated from a theoretical modeling of the structural vibration of the plate is considered for three different excitation frequencies. Data are simulated with the method described in “Simulation of structural vibrations”. Then, the displacement field is converted into phase data and made noisy using a realistic speckle noise simulator^56^. From that the noisy Doppler phases are processed to get the amplitude and phase of the vibration. Three de-noising algorithms are considered: first, the $7 \times 7$ median filter^57^ that is considered for comparison purpose^58^, then the windowed Fourier transform which is a filter operating by applying threshold in the Fourier domain^59^ (noted WFT2F), last a filter operating by Deep Learning approach^60^ (noted DeepL). The way to process the holographic data with the *VibMap* is considered with, first the direct application to noise-free simulated data, second, the direct application to the noisy data, third application to noisy data followed by the de-noising algorithm, and last the de-noising algorithm followed by the application of *VibMap*. Comparison is performed with the exact structural vibration from the simulation and according to the number of periods used for the *VibMap*. Two criteria are used for the comparison: the standard deviation of the error between the processed result and the reference one normalized to the maximum amplitude of the vibration reference, and the MAC criterion between the reference and the reconstructed data. The results are provided in Fig. 3. Similar error analysis can be performed when considering the real holographic measurements and those from the scanning vibrometer. The related curves and plots are provided in Fig. 4.

## Discussion

With Fig. 1, the difference between the spatial resolutions of the two sets of results can be appreciated. Overall, in Fig. 2, the MAC criterion remains quite close to 1 for the diagonal values, thus quantitatively demonstrating that the similarity between results from the scanning vibrometer and those from high-speed coherent imaging is very high. Since the MAC criterion is an indicator which quantitatively appraises the spatial similarities between the two measurement techniques, Fig. 2 shows that holographic measurements are highly coherent with the measurements from LDV overall the studied frequency band. As we can observe in Fig. 3 with the black line curves corresponding to the noise-free data, the algorithm requires only 1 frame to obtain the exact solution. With post-processing operations, the *VibMap* requires relatively few periods to give the correct results and the convergence is obtained with at maximum 10 vibration periods. The standard deviation of the error systematically converges to values less than 10% of the maximum amplitude. It is also important to note that by pre-de-noising with WFT2F and DeepL, this indicator falls down below 5%. For all the methods, the MAC criterion tends to be systematically higher than 0.9, confirming the overall good resemblance of the vibration fields. Note that the pre-de-noising with WFT2F and DeepL provides MAC at almost 0.97 corresponding to faithful retrieval of the vibration. The most efficient method for noise removal is to pre-de-noise the Doppler phases with the WFT2F algorithm before applying the *VibMap*. The DeepL de-noising method yields similar results as WFT2F. Note that at the extreme measurement limits, near to the Nyquist frequency 20kHz, the MAC criterion exhibits a wavy trend that can be interpreted by close under-sampling. Indeed, the measurements are performed with almost 2.015 points per period, the Nyquist criterion is respected, but, periodically, the instant at which the holographic data is recorded will be located close to vibration time nodes, bringing values approximately equal to 0 in the *VibMap*.

The results presented in Fig. 4 overall exhibit the same trends as the one in Fig. 3, even if there are a few discrepancies between the values of the criteria. The standard deviation is overall mainly in the range $[5-10]$% of the maximum value of the reference after reaching the convergence at maximum 20 periods for the high frequency and only a few periods for the low frequency vibrations. The MAC is over 0.9, excepted for the DeepL processing applied after *VibMap* at 19,846.5 Hz. the reason for that is unknown. When the WFT2F, $7 \times 7$ median or DeepL de-noising algorithms are applied before the *VibMap*, then the MAC reaches 0.95. Note that the error computation from experimental measurements requires a point-to-point subtraction between maps. Thus, considering the reduced spatial resolution in the scanning vibrometer measurements, this point-to-point coincidence may be not as accurate as expected. This finally induces a few discrepancies between the values of the criteria in Figs. 4 and  3. Despite this, the criteria indicate that the measurements are very close and that the processing yields very correct estimates of the operational displacement fields when considering $15-20$ periods (depending on the de-noising method used before). In addition, in both the simulation and the measurement cases, the most adapted de-noising method is the WFT2F algorithm very closely followed by the deep learning de-noising method.

The *VibMap* approach can be compared with vibration reconstruction based on Fourier analysis which is more commonly used for processing data from point-wise vibrometers or mechanical accelerometers. The Fourier approach requires a long time-sequence. In the case of holographic measurements, this would require a lot of temporal phase maps, inducing a huge quantity of data to be processed (and also because of the high spatial resolution). Using zero-padding in the Fourier analysis also increases the computation time, even if it increases the accuracy of the peak amplitude estimation. Note also that with Fourier analysis, multiple effects due to windowing appear (typically, convolution with a sinc function) whereas this is not the case with the *VibMap* method. The computation time of the *VibMap* varies linearly with the number of periods while that of Fourier analysis remains constant. As example, for a series of 200 phase maps of $100 \times 100$ pixels, the computation time for *VibMap* is $\sim 0.15 s$ and that of Fourier analysis with padding to 32,768 data points is $\sim 2.7 s$.

## Methods

### Coherent imaging

High-speed coherent imaging based on digital holography provides new opportunities for studying vibrations and acoustic phenomena at both their time and space scales. Digital holograms are produced from the large-field illumination of the object surface to be studied. Since the complex-valued optical field is recorded at any instant, the optical phase, and then, the optical path difference, can be retrieved and yields the measurement of the displacement field at the illuminated surface. Basically, the digital holograms are obtained by recording, with an image sensor organized as a matrix of pixels, the coherent mixing of the diffracted optical wave from the object surface and a known reference wave. If we note *O* the wave front from the illuminated object and *R* the wave front from the reference wave, then the digital hologram can be expressed by Eq. (1)^61,62^:

1 $$\begin{aligned} H=|R|^{2}+|O|^{2}+R^{*} O+R O^{*}. \end{aligned}$$

The reference wave is generally smooth and plane and written $R(x, y)=a_{R} \exp \left[ 2 i \pi \left( u_{0} x+v_{0} y\right) \right]$ with $\left\{ u_{0}, v_{0}\right\}$ its spatial frequencies and $a_{R}$ is a constant. In the proposed approach, the specificity is that the spatial frequencies of *R* are set to $\left\{ u_{0}, v_{0}\right\} = \left\{ 0,0 \right\}$, corresponding to the on-axis configuration. This point is discussed in^49^ and the reader is invited to have a look at the paper for further details. The illuminated object surface is generally at distance $d_0$ from the recording sensor which is used without any imaging lens (arrangement known as the Fresnel configuration). The object wave diffracted to the sensor plane can be expressed with the Fresnel approximations by Eq. (2)^62,63^ ($i = \sqrt{-1}$):

2 $$\begin{aligned} O\left( x, y, d_{0}\right)= & {} -\frac{i}{\lambda d_{0}} \exp \left( \frac{2 i \pi d_{0}}{\lambda }\right) \exp \left( \frac{i \pi }{\lambda d_{0}}\left( x^{2}+y^{2}\right) \right) \nonumber \\&\times \iint A(X, Y) \exp \left( \frac{i \pi }{\lambda d_{0}}\left( X^{2}+Y^{2}\right) \right) \exp \left( -\frac{2 i \pi }{\lambda d_{0}}(x X+y Y)\right) d X d Y . \end{aligned}$$

The object wave front at the object plane is $A(X, Y)=A_{0}(X, Y) \exp \left[ i \psi _{0}(X, Y)\right]$, $\lambda$ is the wavelength of light, $A_0$ is related to the object reflectance and $\psi _{0}$ is the optical phase related to the object surface profile and roughness. From the digitally recorded holograms, the reconstruction of the object field at any distance $d_r$ from the recording plane is given by the discrete Fresnel transform^64^. From the hologram, the numerically reconstructed complex-valued image can be obtained from Eq. (3)^61,62^:

3 $$\begin{aligned} A_{r}=h_{F} \times F F T\left[ H \times h_{F}\right] , \end{aligned}$$

where *FFT* means two-dimensional Fast Fourier Transform and $h_F$ is the Fresnel kernel defined by Eq. (4),

4 $$\begin{aligned} h_{F}(x, y)=\frac{1}{\sqrt{\lambda d_{r}}} \exp \left( i \pi \frac{d_{r}}{\lambda }-i \frac{\pi }{4}\right) \exp \left[ \frac{i \pi }{\lambda d_{r}}\left( x^{2}+y^{2}\right) \right] . \end{aligned}$$

From the numerical computation of Eq. (3), the amplitude and phase of the diffracted field $A_r$ can be evaluated. When the reconstruction distance is $d_r = - d_0$ the initial object plane is recovered and the phase variation from the time sequence is related to the displacement field at the surface. When considering two consecutive time-instants in the hologram sequence, the phase variation is given by Eq. (5) and is similar to the Doppler effect, but from the point of view of the optical phase. The phase change is thus related to the displacement field ${\mathbf {U}}$ rather than the velocity, according to:

5 $$\begin{aligned} \Delta \varphi =\frac{2 \pi }{\lambda } {\mathbf {U}} \cdot \left( {\mathbf {K}}_{e}-{\mathbf {K}}_{o}\right) , \end{aligned}$$

with ${\mathbf {U}} = U_x {\mathbf {i}} + U_y {\mathbf {j}} + U_z {\mathbf {k}}$ ($U_x$, $U_y$ and $U_z$ are the respective displacement fields in the ${\mathbf {i}}$, ${\mathbf {j}}$ and ${\mathbf {k}}$ directions). In Eq. (5), ${\mathbf {K}}_{e}$ is the normalized illumination vector from the light source to the object and ${\mathbf {K}}_{o}$ is the observation vector (also normalized) from the object to the sensor, both defined in a set of reference axis $({\mathbf {i}}, {\mathbf {j}}, {\mathbf {k}})$ attached to the object surface, with ${\mathbf {k}}$ being perpendicular to the surface. In the approach described in this paper, the observation vector is parallel to ${\mathbf {k}}$ and the illumination vector is quasi-oriented along $-{\mathbf {k}}$. Thus, the sensitivity of the phase measurement is oriented along ${\mathbf {k}}$ so that the out-of-plane movement at the surface of the object, $U_z$, can be measured. Generally, the phase variation in Eq. (5) is obtained modulo $2 \pi$ and requires phase unwrapping to yield $U_z$^65^. A particular feature of high-speed digital holography is that thanks to the coherent mixing by heterodyning with the reference wave (Eq. 1), the object wave *O* is amplified by the reference wave *R*, because of the term $R^*O$ included in the recorded hologram (third term of Eq. 1). So, a weak object wave, due to a non-cooperative target, may be balanced by a strong reference wave, if $|R|^{2}>>|O|^{2}$. In addition, the reference wave is directly impacting the sensor and this makes it easier to get large amount of photons for optimizing light detection. In the approach described in this paper, measurements are possible with about 40% of the full sensor dynamics and ratio $|R|^{2} /|O|^{2}$ at about 100, thus yielding suitable phase maps for visualization or metrology purposes.

Note that there are uncertainty sources inducing speckle noise decorrelation in the measured Doppler phase^66^. For example, speckle decorrelation may be due to laser wavelength change between exposures^67^, to reduced number of recording pixels, to defocusing of the reconstructed image^68^ (the reconstruction distance $d_r$ is “not good”), to saturation of the recorded holograms^69^, to quantization with low number of bits, or also due to the extended active surface of pixels (especially for the case of large pixels $> 20 \upmu$ m). Note that different filtering schemes can be applied to de-noise holographic data and exhaustive quantitative comparison was provided in reference^56^. The filtering schemes used in this paper are briefly described further in the text.

### Digital lock-in vibration retrieval

**Figure 5:**
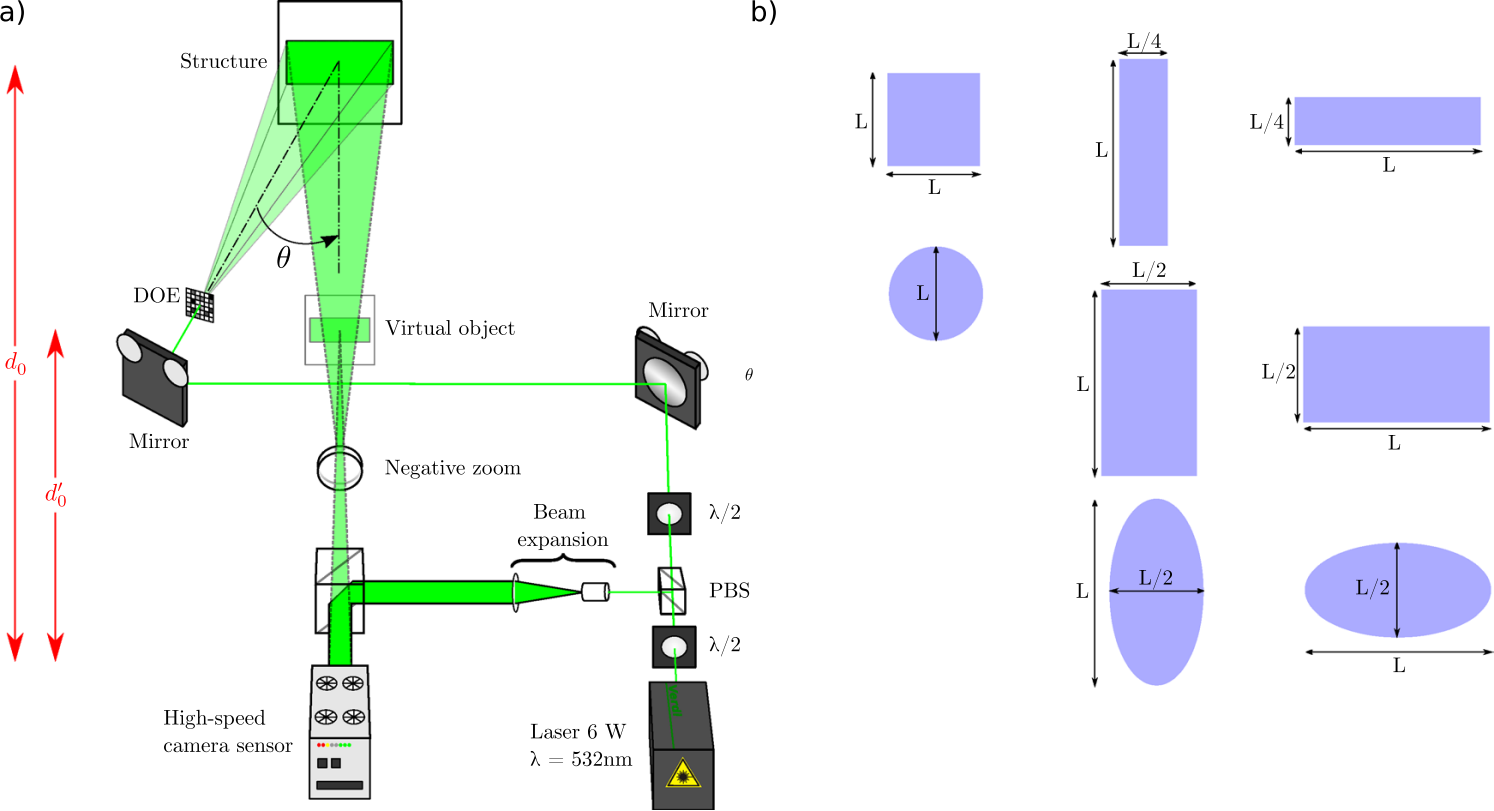
**(a)** Experimental set-up for wide-field holographic vibrometry (PBS: polarizing beam splitter, DOE: Diffractive Optical Element, $\lambda /2$ half-wave plate), **(b)** set of beam shape structures that can be produced by the DOE to illuminate the object surface.

When studying the vibrations of any structure by using a coherent imaging method, one of the characteristic parameters of the recording is the ratio defined by $\alpha =\Delta T / T_{0}$, which is the ratio between the exposure time $\Delta T$ and the vibration period $T_0$. Its value is related to the possibility to reconstruct, without any error, the vibration from a hologram sequence. The second parameter is the cyclic ratio defined as $\beta = T / T_{0}$, which is the ratio between the sensor frame period *T* and the vibration period $T_0$. In this paper, the proposed approach avoids the need for any synchronization between data acquisition and excitation. The only requirement is to know the excitation frequency. This is why the retrieval method is locked on the vibration frequency to process the Doppler phases. When $\alpha<<1$, the recording regime is equivalent of freezing the object at the instant at which the recording is performed. In this case, the optical phase extracted from the reconstructed object field, at any instant $t_1$, is given by,

6 $$\begin{aligned} \psi _{1}=\psi _{0}+\Delta \varphi _{m} \sin \left( \omega _{0} t_{1}+\varphi _{0}+\alpha \pi \right) , \end{aligned}$$

where $\Delta \varphi _{m}$ is the maximum amplitude at pulsation $\omega _0$ (period is $T_0 = 1/f_0$, and $f_0$ the excitation frequency) and $\varphi _{0}$ is the phase of the vibration. The phase $\Delta \varphi _{m}$ is related to the physical vibration amplitude $U_z$ through the sensitivity vector of the optical setup, and the complex-valued displacement field is obtained according to Eq. (7) (for angle $\theta$, see Fig. 5(a)):

7 $$\begin{aligned} {\hat{U}}_{z}= \frac{\lambda \Delta \varphi _{m} }{2 \pi (1+\cos \theta )}\exp {(i \varphi ^{\prime }_0)}. \end{aligned}$$

Let us consider the recording of a time-sequence at sampling rate $f_s=1/T$. Time at the *n*th hologram recording is $t_n = t_1 + (n - 1) T$ and the optical phase difference between two consecutive instants is thus,

8 $$\begin{aligned} \Delta \psi _{n}=\psi _{n+1}-\psi _{n}= 2 \Delta \varphi _{m} \sin (\beta \pi ) \cos \left( \varphi _{0}^{\prime }\right) \cos ((2 n-1) \beta \pi )- 2 \Delta \varphi _{m} \sin (\beta \pi ) \sin \left( \varphi _{0}^{\prime }\right) \sin ((2 n-1) \beta \pi ). \end{aligned}$$

Here, $\varphi _{0}^{\prime }=\omega _{0} t_{1}+\varphi _{0}+\alpha \pi$. Finding $\Delta \varphi _{m}$ and $\varphi '_0$ from Eq. (8) can be done in the least square sense. Equation (8) can be rewritten with matrix notations where matrix ${ {X}}$ includes the known theoretical coefficients, vector ${\Delta \psi }$ includes the measured Doppler phases and vector ${{a}}$ includes the unknown to be determined. We have $${{a}}= \left[ \begin{array}{cc} a_1&a_2 \end{array}\right] ^T$$ (upper script *T* meaning transpose matrix), with $a_{1} = 2 \Delta \varphi _{m} \sin (\beta \pi ) \cos \left( \varphi _{0}^{\prime }\right)$ and $a_{2}=2 \Delta \varphi _{\text{ m } } \sin (\beta \pi ) \sin \left( \varphi ^{\prime }_0\right)$. Matrix ${ {X}}$ is described by Eq. (9) where $n_s$ is the number of digital holograms in the recorded time sequence:

9 $$\begin{aligned} { {X}}= \left( \begin{array}{cc} \cos (\beta \pi ) &{} -\sin (\beta \pi ) \\ \cos (3\beta \pi ) &{} -\sin (3\beta \pi ) \\ \cos (5\beta \pi ) &{} -\sin (5\beta \pi ) \\ \cdots &{} \cdots \\ \cdots &{} \cdots \\ \cdots &{} \cdots \\ \cos ((2n_s-1)\beta \pi ) &{} -\sin ((2n_s-1)\beta \pi ) \end{array}\right) . \end{aligned}$$

The mean square estimation of ${ {a}}$ is deduced from the cost function ${ {J}}=( {\Delta \psi }-{ {X}} { {a}})^T{ {I}}( {\Delta \psi }-{ {X}} { {a}})$ when calculating $\partial {{ {J}}} / \partial {{ {a}}}$, which yields the optimal solution ${a_{opt}}=({ {X}}^T { {I}} { {X}})^{-1}{ {X}}^T { {I}} {\Delta \psi }$, where ${ {I}}$ is the identity matrix. Finally, one gets $\varphi ^{\prime }_0=\tan ^{-1}\left( a_{2} / a_{1}\right)$ and $\Delta \varphi _{m}=\sqrt{\left( a_{1}^{2}+a_{2}^{2} \right) } / 2 \sin (\beta \pi )$. So, the amplitude and the phase (to a nearest non essential constant) of the vibration can be measured from the hologram sequence. In the paper, this algorithm for amplitude and phase retrieval of the structural vibration is called according to the acronym *VibMap*.

### Experimental set-up

The experimental set-up is described in Fig. 5a). The light is emitted from a continuous wave laser at $\lambda = 532$ nm with maximum power of 6W. The half-wave plate at the output of the laser is used to adjust the power in both object and reference paths to get an adequate $|R|^{2} /|O|^{2}$ ratio. The laser is separated into a reference wave and an object wave by use of a polarizing beam splitter (PBS). The polarization of the object wave is then rotated $90^{\circ }$ to be parallel with that of the reference wave, so that interferences may occur. The reference wave is expanded, spatially filtered using a spatial filter (microscope objective and microscopic pinhole), and collimated to produce a smooth plane reference wave impacting the sensor at normal incidence. So, the set-up is arranged in the on-line configuration^49^.

The object wave is spatially expanded to illuminate the structure by using a dedicated DOE (Diffractive Optical Element)^70^. The DOE was designed with 8 subareas, each of them producing a particular laser beam shape^71^. Figure 5 b) illustrates the diversity of shapes that can be produced with the DOE: square area, elliptical areas, narrow and large rectangular beams (vertical and horizontal). Such beam shaping increases the photometric efficiency of the set-up by avoiding wasting light with classical lenses and mirror assembly. In the set-up, the illumination angle is about $\theta = 15 ^{\circ }$. The object and reference waves are combined by the 50% beam splitter cube placed just in front of the high-speed sensor. In the optical path from the object surface to the sensor plane, a negative zoom is inserted in front of the cube. This negative zoom produces a smaller image of the object when reducing the object-to-sensor distance^72,73^. This has the advantage of producing a smaller virtual object facing the sensor at smaller distance $d'_0$. In this way, the dimensions of the virtual object are compatible with the requirement from the Shannon conditions for recording digital holograms^62^. Basically, from the hologram, the virtual image can be computed when setting $d_r = -d'_0.$ The physical object plane is obtained by correctly scaling the set of reference coordinates attached to the image plane. If one notes $g_{opt}$ the optical magnification ($0< g_{opt} < 1$) produced by the negative zoom, then the spatial resolutions in the final image are given by:

10 $$\begin{aligned} \left\{ \begin{array}{c} \rho _{x}=\frac{\lambda d_{0}}{N p_{x} g_{opt}} \\ \rho _{y}=\frac{\lambda d_{0}}{M p_{x} g_{opt}}. \end{array}\right. \end{aligned}$$

The sensor is a high-speed camera from Photron, with pixel pitch at $p_x = p_y =20\,\upmu$m and maximum spatial resolution of $1024 \times 1024$ pixels. At the full spatial resolution, the maximum frame rate is 12,500 Hz. When increasing the frame rate, the spatial resolution is degraded, that is $328 \times 768$ at 50 kHz and $264 \times 384$ at 100 kHz. The exposure time can be set from 380 ns to few ms. In this paper, the exposure time was set at 1 $\upmu$s and the laser power was adjusted at 3 W.

**Figure 6:**
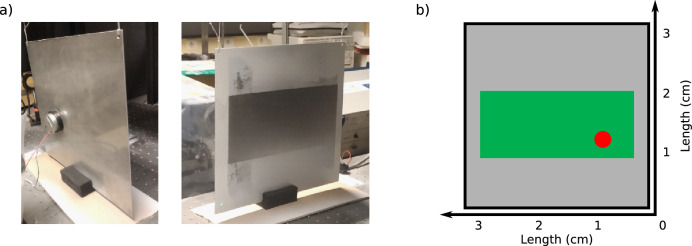
**(a)** Photographs of the set-up with the plate clamped to the bench and the mechanical shaker, **(b)** scheme of the plate, green is the zone measured by holography and red spot indicates the location of the excitation source.

The studied structure is a steel plate from dimension $30 \times 30$cm$^2$ steel plate 2mm thick. Figure 6a shows the photograph of the assembly with the plate and the excitation set-up. A schematic representation of the plate and the location of the excitation point can be seen in Fig. 6b. Vibrations of the structure are generated by an electro-mechanical loudspeaker glued to the structure. A piece of foam is placed under the plate to suppress the pendulum movement of the free structure.

When adjusting the negative zoom to capture holograms from a rectangular area sized 25.6 cm $\times$ 12.6 cm (about 322.56 cm$^2$), the focal length was set to − 75 mm, leading to the reconstruction distance at $d'_0 = -300$ mm. In the set-up, the distance between the initial object plane and the sensor plane is about 263.5cm and the optical magnification is $g_{opt} \sim 0.0294$. The digital holograms are recorded at spatial resolution of $512 \times 512$ pixels, and with the experimental parameters the spatial resolutions in the reconstructed image are respectively given along the *x* and *y* directions by $\rho _{x} = \rho _{y} \simeq 464.04 \upmu \text {m}$.

### Simulation of structural vibrations

The simulated operational displacement fields used for the quantitative appraisal of *VibMap* are calculated with a method based on forced wave decomposition. The main advantage compared to a modal decomposition approach is the calculation of the exact response of the system and thus this does not suffer from any mode truncation. Note that the forced wave synthesis method is useful when the spatial resolution is very high (i.e. the spatial resolution limits will not cut wave numbers). The theoretical expression can be easily obtained in the case of 1D structures (in the case of beam for example) but becomes more difficult to calculate with two dimensions. In this case, the forced response is calculated from simple series combining modal synthesis in one direction and forced wave response in the other direction. It will then be possible to limit the impact of truncation by considering a large number of modes (this expression requires fewer resources to be computed). The expression of the out-of-plane displacement field $U_z$ for a simply supported plate is given by^74^:

11 $$\begin{aligned} {\hat{U}}_z(x,y,\omega ) = \left\{ \begin{array}{l} \sum _{n = 1}^{\infty } \left( A_{1n} \sin (k_n y) + c_{1n}\sinh (\gamma _n y) \right) \sin \left( \frac{n \pi }{L_x} x \right) , \text{ for } y \in \left[ 0 ; y_{0}\right] \\ \sum _{n = 1}^{\infty } \left( A_{2n} \sin (k_n (y - L_y)) + c_{2n}\sinh (\gamma _n (y - L_y)) \right) \sin \left( \frac{n \pi }{L_x} x \right) , \text{ for } x \in \left[ x_{0} ; L\right] \end{array}\right. \end{aligned}$$

In Eq. (11), $k_n = \sqrt{\sqrt{\frac{\rho h \omega ^2}{D}} - \left( \frac{n \pi }{L_x} \right) ^2}$ and $\gamma _n = \sqrt{\sqrt{\frac{\rho h \omega ^2}{D}} + \left( \frac{n \pi }{L_x} \right) ^2}$ are respectively the number of progressive waves and the number of evanescent waves while *h* is the thickness, *D* the stiffness of the structure and $\omega = 2\pi f_0$ with $f_0$ the excitation frequency. The coefficients $A_{1n}$, $C_{1n}$, $A_{2n}$ and $C_{2n}$ are obtained by inverting the system of equations given in Eq. (12):

12 $$\begin{aligned} \left( \begin{array}{cccc} \sin (k_n y_0) &{} \sinh (\gamma _n y_0) &{} -\sin (k_n (y_0 - L_x)) &{} -\sinh (k_n (y_0 - L_x)) \\ k_n\cos (k_n y_0) &{} \gamma _n \cosh (\gamma _n y_0) &{} -k_n \cos (k_n (y_0 - L_x)) &{} -\gamma _n\cosh (\gamma _n (y_0 - L_x)) \\ -k_n^2\sin (k_n y_0) &{} \gamma _n^2\sinh (\gamma _n y_0) &{} k_n^2\sin (k_n (y_0 - L_x)) &{} -\gamma _n^2\sinh (k_n (y_0 - L_x)) \\ -k_n^3\cos (k_n y_0) &{} \gamma _n^3 \cosh (\gamma _n y_0) &{} k_n^3 \cos (k_n (y_0 - L_x)) &{} -\gamma _n^3\cosh (\gamma _n (y_0 - L_x)) \end{array}\right) \left( \begin{array}{c} A_{1n} \\ C_{1n} \\ A_{2n} \\ C_{2n} \end{array}\right) = \left( \begin{array}{c} 0 \\ 0 \\ 0 \\ \frac{-2 A_0}{D L_x} \sin \left( \frac{n \pi }{L_x}x_0\right) \end{array}\right) , \end{aligned}$$

where the source is localized at coordinates $[x_0,y_0]$ at the plate sized $[L_x,L_x]$. The inversion of the system is made with the *Symbolic Math Toolbox* from Matlab$\copyright$ environment. The simulated time-dependent out-of-plane vibration is then obtained with (${\mathbb {R}}$ means real part of the complex valued data):

13 $$\begin{aligned} U_z(x,y,t) = {\mathbb {R}}\left\{ {\hat{U}}_z(x,y,\omega ) \exp \left( i \omega t \right) \right\} . \end{aligned}$$

The time-varying vibration maps can be obtained for a chosen sampling frequency $f_s$ by defining the discrete time axis as $t_n = \frac{n}{f_s}$. Simulations are carried out with conditions close to that of experimental measurements in order to get realistic vibration patterns. The simulated aluminum plate has dimensions of $L_x \times L_y=30 \times 30$cm$^2$, thickness *h*=2mm, Young’s modulus is $E=70GPa$, density is $\rho =2.7 \times 10^{-3}kg/m^3$, Poisson’s coefficient $\nu =0.33$ and the loss factor is $\eta _E=1 \times 10^{-3}$. The excitation is localized at $[x_0, y_0] = [20,20]$cm. The useful rectangular area cut out is limited to x $\in$ [2-28]cm and y $\in$ [10-20]cm. The excitation amplitudes are 3N, 5.5N and 0.25N respectively for 6353.5Hz, 14858Hz and 19846.5Hz.

### Realistic simulation of noise in vibration phase data

**Figure 7:**
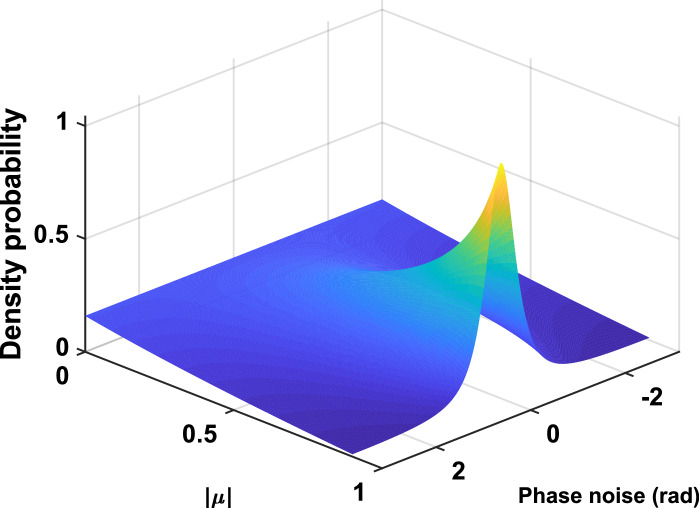
Map of the probability density functions of the speckle noise for different values of the modulus of the coherence factor $| {\mu }|$.

In digital holographic interferometry, the corruption of phase data is mainly due to speckle phase decorrelation. The decorrelation noise can be realistically simulated by considering experimental conditions as described in Ref.^56^. The deformation due to the vibration, $U_z(x,y,t_n)$, is put into the noise simulator, then the noisy Doppler phases are obtained by calculating the difference of the successive output noisy maps from the algorithm. As results, the simulator yields speckle phase decorrelation noise having the same statistics as that estimated from real experimental phase maps. The probability density function of phase noise $\epsilon$ is the given by the following equation^75^:

14 $$\begin{aligned} p(\epsilon ) = \frac{1-| {\mu }|^2}{2\pi }\left( 1-\beta ^2\right) ^{-3/2}\left( \beta \sin ^{-1}(\beta )+\frac{\pi \beta }{2}+\sqrt{1-\beta ^2}\right) , \end{aligned}$$

with $\epsilon$ the phase noise, $\beta = | {\mu }|\cos (\epsilon )$ and $| {\mu }|$ the modulus of the complex coherence factor of the speckle field between the two considered instants. The decorrelation noise is related to the signal-to-noise ratio (SNR) in the phase map from high SNR with $| {\mu }| \sim 1$ to low SNR with $| {\mu }| \sim 0$. The probability density functions for different values of the modulus of the coherence factor are plotted in Fig. 7. Note that the speckle decorrelation noise is non Gaussian and non stationary, because it depends on the vibration amplitude. The local noise statistics depend on the local deformation amplitude. For simulations, the speckle size can be adjusted to be as close as possible to that of the real experiments.

### De-noising algorithms

Data were processed using three de-noising methods which are briefly summarized here after.

#### Median filter

The median filter is considered as a reference filter^57^ for test and comparison of more advanced de-noising strategies. The main parameter is the window size and it is therefore advisable to choose that to be used wisely. In this paper, the window size has been set to 7 in order to get efficient filtering and to reduce the artifacts at edges of the data maps.

#### Windowed Fourier transform filter (WFT2F)

This algorithm was designed for de-noising phase fringe patterns from speckle interferometry^59^ . It was demonstrated to be the highest performing algorithm for phase data from digital holographic interferometry^56,58^. This algorithm reduces the speckle noise in the frequency domain by using local Fourier transforms which can take the non-stationary characteristics of the noise into account. Threshold applied to the modulus of the 2-D Fourier coefficients, having the phase unchanged, is performed and an inverse 2-D windowed Fourier transform of the filtered frequency domain is then computed to get the modulo $2\pi$ phase data. The threshold permits to retain high-frequency information while eliminating residual noise independent of frequency.

#### De-noising with deep learning

Since about a decade, machine learning, and more precisely deep learning, has emerged as a very efficient tool in signal and image processing. At the heart of this new tool is the convolutional neural network (CNN) which integrates several fundamental advances of last decades: wavelet and multiresolution analysis, shrinking and thresholding algorithms, sparse representations, block matching and dictionary learning^76,77^. In recent years, several applications of deep learning in optics have emerged, the deep learning approach is applied to noise reduction and to enhance the quality of the reconstructed tomographic image quality^60,78,79^. In previous works, the problem of speckle decorrelation noise was approached with a deep learning based solution^60^ exhibiting performances at the state-of-the art for speckled phase data de-noising. The network based on residual learning^80^ has been adapted to be trained with a set of fringe patterns including realistic speckle noise conditions, similarly as what is usually encountered in coherent phase imaging. Results show that the deep learning approach achieved comparable performances in terms of standard deviation of phase error to the 2D-Windowed Fourier method but with better algorithmic efficiency using GPU architecture.

### Criteria for error appraisal

For the quantitative appraisal of errors, criteria were considered in order to analyze the ability of the algorithms to retrieve the amplitude and phase of the operational deformations of the structure. The first criterion is the standard deviation of the error between the processed amplitude and a reference one (simulated or measured by pointwise vibrometer). The standard deviation is normalized to the maximum amplitude of the reference. The second criterion is the Modal Assurance Criterion^81,82^, MAC, and its related derivatives^83^. The MAC is based on the principle of orthogonality of the modes of any structure. If these modes are completely different, the scalar product of these two modes will be zero, so the MAC tends to 0, and in the case of perfect resemblance it will tend to 1. The MAC criterion is defined for a set $p,q \in (1,n_{op})$ of $n_{op}$ operational deformations, organized as column vector, where $\{\Phi _A\}$ are measured with instrument/method *A* and $\{\Phi _B\}$ with instrument/method *B*, as follows:

15 $$\begin{aligned} MAC(p,q) = \frac{\{\Phi _A\}_p^T \{\Phi _B\}_q}{\left( \{\Phi _A\}_p^T \{\Phi _B\}_p\right) \left( \{\Phi _A\}_q^T \{\Phi _B\}_q\right) }. \end{aligned}$$

In this way, when comparing an entire set of $n_{op}$ operational deformations, only modes exhibiting similarities between the two measurement/calculation methods will yield MAC close to 1. The graphical representation of the MAC is similar to a correlation matrix as depicted in Fig. 2.

### Schemes for data processing

**Figure 8:**
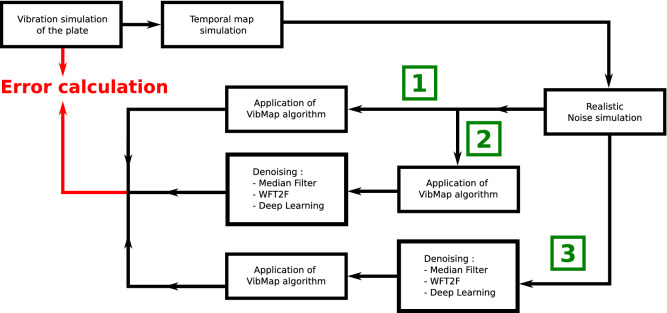
Different schemes for the simulation chain for data obtained from realistic simulations, green numbers 1, 2, 3 indicate the three ways for the data processing.

This section is devoted to the methodology in order to evaluate the performance of the processing. The principle is to use realistic simulated data starting with simulation of a vibrating plate. Then, the speckle noise is added to the data using realistic noise simulation^56^. There are three ways for applying the *VibMap* process. The first is the direct application of the algorithm to the raw data; the second is the application of *VibMap* followed by de-noising, and the third is the de-noising step followed by the *VibMap* algorithm. So, considering the 3 de-noising algorithms, there are 9 possibilities of using the *VibMap* when considering those different filtering schemes. Figure 8 illustrates the full simulation chain with the three options. At the end, one is able to calculate the error between what was initially simulated and what is provided by the full data processing. In order to mimic the real experimental on-line holographic arrangement, the speckle grain was adjusted to 1.3 pixels. The decorrelation noise amount is automatically adjusted by the vibration amplitude and has non Gaussian statistics and is not stationary. The main parameter to characterize the decorrelation noise is the modulus of the coherence factor, $| {\mu }|$, which is an excellent quality indicator. If $| {\mu }| < 0.85$ the data are considered as highly noisy and processing may be difficult, whereas when $| {\mu }| \ge 0.85$, the measurements have the requested quality. Results of the error investigation with simulated data are given in Fig. 3 where the standard deviation and the MAC criterion are represented for the three simulated frequencies versus the number of periods in the *VibMap*.

**Figure 9:**
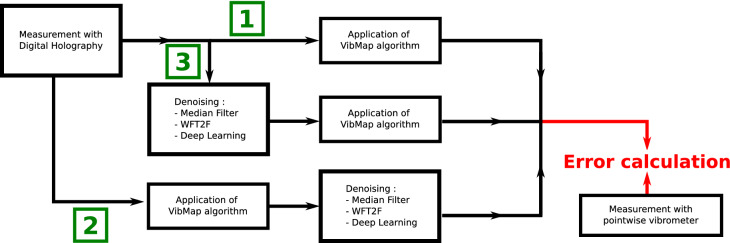
Three schemes for the error evaluation from experimental data, green numbers 1, 2, 3 indicate the three ways for the data processing.

In the same way as discussed for Fig. 8, the approach for error investigation can be carried out with the experimental results from the scanning laser vibrometer and those from coherent imaging. The scheme for the measurement chain is shown in Fig. 9 and results are given in Fig. 4.
